# Cutaneous Manifestations of Lightning Injury: A Case Report

**Published:** 2008-09-16

**Authors:** M. E. Asuquo, I. A. Ikpeme, I. Abang

**Affiliations:** Department of Surgery, University of Calabar Teaching Hospital, Calabar, Nigeria

## Abstract

**Background:** Lightning injuries are relatively uncommon and have been a subject of awe since primitive times. It most significantly affects the cardiorespiratory, nervous, and integumentary systems. Surprisingly, cutaneous burn injuries caused by lightning are usually superficial. **Objective:** To present the cutaneous manifestations of lightning injuries and the sequelae of improper management. **Case report:** A 22-year-old woman presented with cutaneous manifestations of lightning-induced burns and bilateral upper limb gangrene after 2 months of improper treatment. She refused amputation after counseling and left the hospital. **Conclusion:** This is a rare case of burns with cutaneous manifestations peculiar to lightning injury. These features serve as evidence of lightning injury, when in doubt, especially in societies where superstition is rife. Education concerning the nature of lightning and proper management would improve outcome.

Injuries from man-made, generated, or technical electricity have been reported for only about 150 years, but injuries from lightning surely predate written records. Over the centuries, superstitions and myths about lightning have grown.^1^ Its power has been a subject of awe since primitive times. It is a natural atmospheric electrical discharge that occurs between regions of net positive and negative electric charges.^2^ Lightning injuries are commoner in rural or exposed environments than in the city where high buildings have metal frames and lightning protection devices. It has been estimated that lightning strikes have a 30% mortality rate.^3^ Most significantly affected are the cardiorespiratory, nervous, and integumentary systems.^4^ Skin injuries vary from cutaneous injury to that typically caused by high-voltage, commercial electricity. Injury is affected by the type of clothing, amount of moisture on the skin, and the presence of metal on the body. Burn injuries are surprisingly superficial.^2^ We present this report to highlight the importance of appreciating the various cutaneous manifestations of lightning injury, including the complications of improper management.

## CASE REPORT

A 22-year-old woman with a history of inability to use both upper limbs since 2 months presented in the accident and emergency unit. This followed a lightning strike in a village while she was asleep in the house. She was partially clothed with the trunk exposed during the incident. There was no history of loss of consciousness. Following the injury, she sought treatment from a traditional/spiritual healer in view of the superstitious belief of being attacked by evil forces.

**Figure 1:**
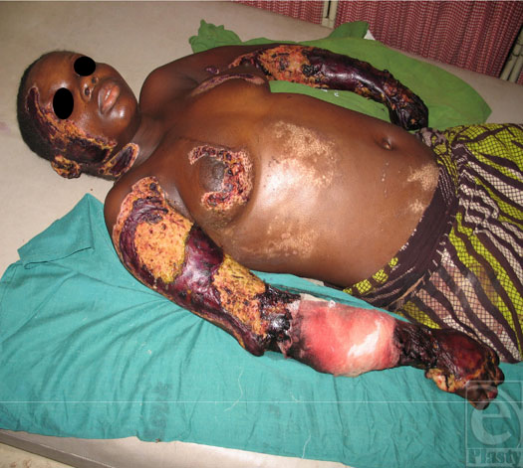
Cutaneous manifestation of lightning injury with bilateral below-elbow gangrene.

On examination, she was in poor general condition, febrile, and anemic. She had healing/ or infected superficial burns that involved the right side of the face and neck, the right axilla, breasts, right hypochondrium, and iliac fossa. The lower extremities of the upper limbs had extensive gangrene that extended to the elbows and infected burns on the right arm extending to the deltoid region as well as the anterior part of the left arm (Figure 1). On examination, chest and abdomen as well as the lower limbs were normal.

A diagnosis of 18% lightning-induced burns with bilateral below-elbow gangrene was made. She was resuscitated with intravenous fluids, antibiotics, and immunized against tetanus. The patient refused amputation after counseling and left the hospital.

## DISCUSSION

Lightning strike is rare and causes human injury by 4 distinct mechanisms: direct, splash, stride, and blast effect. The type of injury depends on energy imparted to the body by the strike that may be mechanical, thermal, or electrical.^4^ Lightning injuries may be classified into minor (no loss of consciousness with or without amnesia), as was the case in our index patient; moderate (loss of consciousness and subsequent recovery with minor deficit); and severe (full cardiopulmonary arrest).^5^,^6^

Most significantly affected by lightning strikes are the cardiorespiratory, nervous, and integumentary systems, the latter of which is the focus of this discussion. There are 6 types of burns caused by lightning: feathering, linear, punctuate, thermal, contact, and flash burn. Lightning strikes cause characteristic cutaneous effects like dermal ferning or feathering that is an aborescent pattern to the skin and, if found in a comatose patient, is presumptive evidence of a lightning strike.^7^,^8^ The effects disappear after several days.

Although the temperatures associated with thermal injury are extremely high, the heat is rapidly dissipated and most resultant skin burns are superficial with little effect on deep tissue.^9^ Our patient presented with first- and second-degree burns beneath and around the breasts, right side of the face, ear, neck, chest wall, and lower abdomen (Figure 1), and these were flash burns. However, when mismanaged and infection ensues, the injuries may progress to deeper dermal layers.

Linear burns are partial thickness burns that occur over moisture rich areas of the body, such as beneath the breasts, down the mid chest, and the midaxillary line (Figure 1). They are present minutes to hours after injury and result from vaporization of sweat into steam on the patients' body.^2^ The resistance of wet skin is lower, and lightning travels on the surface without any penetration—“flashover effect.”^4^

Our patient presented with full-thickness infected burns and bilateral gangrene extending to the elbows. The burn injuries became infected overtime and worsened during the period of topical herbal treatment, in view of the history of malodorous discharge with progressive inability to use the limbs. Full-thickness burns rarely result from lightning accidents. However, occasionally there may be an electric burn from direct current flow with clinical manifestation similar to those from a commercial high-voltage electrical injury. A thermal burn results when lightning ignites clothings.

In societies where lightning is not regarded as natural, the traditional healer is usually the first to be consulted. This patient was managed in such facility for 2 months prior to presentation. Therapy for burn injuries includes debridement followed by application of topical antimicrobial agents; we use topical honey. Tetanus prophylaxis is mandatory. When full-thickness burns are evident, excision and autogenous split-thickness skin graft are offered treatment options. Infection from poor wound care may result in the injury worsening and when limbs are involved, gangrene may ensue necessitating limb ablation or death from septicemia, as depicted in this patient. In the absence of gangrene, extensive burn scars may develop, after many years, into squamous cell carcinoma—Marjolin's ulcer—in poorly managed cases.^2^

Burns location provides a prognostic indicator. Cranial and lower extremity burns have a 4- and 5-fold increase in mortality, respectively, due to such burns in any other location.^10^ However, irrespective of location, poor wound management resulting in infection and gangrene is a further indicator of increase in morbidity and mortality, as depicted in this case study.

In conclusion, this patient demonstrated striking integumentary manifestations of lightning injury, which progressed to gangrene during topical herbal treatment that accounted for late presentation with poor outcome. Lightning is a natural phenomenon; early presentation with prompt and proper care would engender an improved outcome.
